# High sensitivity troponin T and I reflect mitral annular plane systolic excursion being assessed by cardiac magnetic resonance imaging

**DOI:** 10.1186/s40001-017-0281-x

**Published:** 2017-10-04

**Authors:** Michèle Natale, Michael Behnes, Seung-Hyun Kim, Julia Hoffmann, Nadine Reckord, Ursula Hoffmann, Johannes Budjan, Siegfried Lang, Martin Borggrefe, Theano Papavassiliu, Thomas Bertsch, Ibrahim Akin

**Affiliations:** 1 First Department of Medicine, Faculty of Medicine Mannheim, University Medical Center Mannheim (UMM), University of Heidelberg, Theodor-Kutzer-Ufer 1-3, 68167 Mannheim, Germany; 2 Institute of Clinical Radiology and Nuclear Medicine, Faculty of Medicine Mannheim, University Medical Center Mannheim (UMM), University of Heidelberg, Mannheim, Germany; 3 Institute of Clinical Chemistry, Laboratory Medicine and Transfusion Medicine, General Hospital Nuremberg, Paracelsus Medical University, Nuremberg, Germany

**Keywords:** Mitral annular plane systolic excursion, Natriuretic peptide, NT-proBNP, High sensitivity troponin, troponin I, troponin T, Magnetic resonance imaging, MRI

## Abstract

**Purpose:**

This study aims to evaluate the association between high sensitivity troponins (hsTn) and mitral annular plane systolic excursion (MAPSE) in patients undergoing cardiac magnetic resonance imaging (cMRI).

**Methods:**

Patients undergoing cMRI were prospectively enrolled. Patients with right ventricular dysfunction (< 50%) were excluded. Blood samples for measurements of hsTn and amino-terminal pro-brain natriuretic peptide (NT-proBNP) were collected at the time of cMRI.

**Results:**

84 patients were included. Median left ventricular ejection fraction was 59% (IQR 51–64%). HsTn were correlated inversely with MAPSE within multivariable linear regression models (hsTnI: Beta − 0.19; *T* − 1.96; *p* = 0.05; hsTnT: Beta − 0.26; *T* − 3.26; *p* = 0.002). HsTn increased significantly according to decreasing stages of impaired MAPSE (*p* < 0.003). HsTn discriminated patients with impaired MAPSE < 11 mm (hsTnT: AUC = 0.67; *p* = 0.008; hsTnI: AUC = 0.64; *p* = 0.03) and < 8 mm (hsTnT: AUC = 0.79; *p* = 0.0001; hsTnI: AUC = 0.75; *p* = 0.001) and were still significantly associated in multivariable logistic regression models with impaired MAPSE < 11 mm (hsTnT: OR = 4.71; *p* = 0.002; hsTnI: OR = 4.22; *p* = 0.009).

**Conclusions:**

This study demonstrates that hsTn are able to reflect MAPSE being assessed by cMRI.

**Electronic supplementary material:**

The online version of this article (doi:10.1186/s40001-017-0281-x) contains supplementary material, which is available to authorized users.

## Background

Mitral annular plane systolic excursion (MAPSE) represents a central parameter of cardiac function being measured by cardiac magnetic resonance imaging (cMRI) [1]. MAPSE correlates with systolic longitudinal left ventricular (LV) contractility, and thus complements the assessment of LV ejection fraction (EF), which itself reflects circumferential LV contractility [2]. A reduction of MAPSE was shown to correlate with the presence of heart failure (HF) with preserved ejection fraction (HFpEF) [3]. Moreover, it was demonstrated that reduced MAPSE might reflect increased cardiac mortality and re-hospitalization incidence in patients with HF, as well as in those with atrial fibrillation (AF) and after myocardial infarction [4].

Cardiac biomarkers, such as natriuretic peptides, reveal powerful diagnostic and prognostic values for the risk-stratification of patients with HF [5]. Increased levels of amino-terminal pro-brain natriuretic peptide (NT-proBNP) were shown to be associated with MAPSE as being evaluated by transthoracic echocardiography [6]. Cardiac troponins (cTn) represent the major enzymes for myocardial contractility [7]. Recently developed sensitive troponin assays are characterized by an approximately hundredfold lower detection limit than conventional troponin assays [8]. Therefore, measurements of cardiac high sensitivity (hs) troponin I (TnI) and T (TnT) might bear the potential to diagnose a myocardial infarction much earlier compared to conventional troponin tests [9, 10].

Within a community registry of atherosclerosis, elevated hsTn were shown to reflect the presence of chronic heart failure (CHF) better than conventional tests due to its lower detection limit [11]. Furthermore, cardiac biomarkers, such as NT-proBNP and hsTn, were shown to be associated with short- and long-term-prognosis in patients suffering from CHF with reduced and preserved ejection fraction (HFrEF and HFpEF) [7, 12, 13]. In addition, it was demonstrated that the concentration of hsTn related significantly to LVEF and LV end-diastolic pressure measured by transthoracic echocardiography [14]. However, whether hsTn might be able to reflect MAPSE being assessed by cMRI is hardly investigated.

Therefore, this study aims to investigate whether concentrations of hsTn are able to reflect MAPSE being assessed by cMRI.

## Methods

### Study population

The “Cardiovascular Imaging and Biomarker Analyses” (CIBER) study (clinicaltrials.gov identifier: NCT 03074253) represents a clinically prospective, controlled and monocentric study conducted at the University Medical Center Mannheim, Germany. The research adhered to the principals outlined in the Declaration of Helsinki and was approved by a regional ethics committee. Written informed consent was obtained from all patients.

For the present study, patients undergoing cMRI during routine clinical care were included consecutively to this study from February 2015 until June 2015 within an all-comers design. To perform valuable cMRI examination all patients had to be in a stable clinical condition without acute clinical symptoms, such as acute dyspnea or extensive peripheral edema. The indications for cMRI were not restricted to any specific cardiac disease entity. Exclusion criteria for cMRI accorded to commonly known exclusion criteria, such as claustrophobia and metal implants [1]. Specifically for the present study, patients with a reduced right ventricular function (RVF) below 50% were excluded.

All available clinical information of the study patients were documented, such as detailed findings of patients’ prior medical history, laboratory findings and medical therapies. Blood samples for biomarker measurements were collected once within 24 h following cMRI examination.

### Measurements of biomarkers

All expressed biomarkers were measured in the serum of patients’ blood. All samples were obtained by venipuncture into serum monovettes^®^ and centrifuged at 2000 *g* for 10 min at 20 °C. The aliquoted samples were cooled down with liquid nitrogen before being stored at − 80 °C until analysis. The complete processing was conducted within two hours after blood extraction. After thawing, the samples were mixed gently by inverting and centrifuged with 2500*g* for 10 min at 20 °C, respectively, 3000*g* for 30 min for hsTnI at 4 °C.

HsTnT was measured with the Troponin T hs STAT assay on a cobas e 602 analyzer (Roche Diagnostics, Mannheim, Germany). The limit of blank (LoB) for this assay was 3 ng/L and the limit of detection (LoD) was 5 ng/L as described in the instructions for use [15]. HsTnI was measured with the STAT High sensitivity Troponin-I assay on an Architect i1000 analyzer (Abbott, Wiesbaden, Germany). The LoB was 0.7–1.3 ng/L and the LoD was 1.1–1.9 ng/L for this assay as described in the instructions for use [16]. NT-proBNP was measured with the proBNP II STAT assay on a cobas e 602 analyzer (Roche Diagnostics, Mannheim, Germany). The LoD for this assay was 5 ng/L [17]. Creatinine was measured with the Creatinine Jaffe Gen.2 assay on a cobas c 702 analyzer (Roche Diagnostics, Mannheim, Germany).

### cMRI acquisition

All studies were performed using a 1.5-T whole-body imaging system (Magnetom Avanto and Sonata, Siemens Medical Systems, Healthcare Sector, Erlangen, Germany) using a four-element (Sonata) or six-element (Avanto) phased-array body coil. Cine images were acquired using a retrospective electrocardiographic-gated, balanced segmented steady-state free precession (trueFISP) sequence in three long-axis views (2-, 3-, and 4-chamber views) and in multiple short-axis views, covering the entire left ventricle from base to apex.

### cMRI analysis

The cMRI image analysis was performed using the commercially available computer software program cvi^42®^ (Circle Cardiovascular Imaging Inc., Calgary, Canada). MAPSE measurements were assessed on four-chamber view cine images. The distance between the basal septal mitral annulus (septal MAPSE), the basal lateral mitral annulus (lateral MAPSE) and a reference point outside the left ventricular apex was measured in end-diastole and end-systole. The distance traveled by the septal and lateral annulus from end-diastole to end-systole was calculated as septal and lateral MAPSE by subtracting the left ventricular end-systolic length from the left ventricular end-diastolic length as being described previously [18]. Average MAPSE was calculated as the average of septal and lateral MAPSE. Three sub-groups were set according to MAPSE: (MAPSE I: ≥ 11 mm, MAPSE II: ≥ 8 to < 11 mm, MAPSE III: < 8 mm).

### Statistical analysis

For data with normal distribution, the Student’s t test was applied. Otherwise, Kruskal–Wallis test was used as non-parametric test. Deviations from a Gaussian distribution were tested by the Kolmogorov–Smirnov test. First, the clinical confounding factors influencing MAPSE in the total cohort were evaluated within multivariable linear regression model adjusted for hsTn and clinical parameters or comorbidities (coronary artery disease (CAD), valvular heart disease and AF). Second, MAPSE subgroups were set into MAPSE I: ≥ 11 mm; MAPSE II: ≥ 8 to < 11 mm; MAPSE III: < 8 mm and the distribution of cMRI indices according to MAPSE subgroups was analyzed. Third, univariate correlations between hsTn and cMRI parameters in all patients were analyzed using Spearman’s rank correlation for non-parametric data. In a fourth step, multivariable linear regression models adjusted for basic parameters (age, sex, creatinine) and clinical parameters or cardiac comorbidities (CAD, valvular heart disease, AF, impaired left atrial function (LAF) < 45% and impaired MAPSE < 8 mm) were performed for evaluating influencing factors on hsTn in the present cohort. Thereafter, receiver operating characteristic (ROC) curve analyses with area under the curves (AUC) were determined to evaluate whether biomarkers are able to discriminate the presence of reduced MAPSE. ROC curves were compared by the method of Hanley et al. [19]. In a last step, multivariable logistic regression models were developed to confirm the diagnostic value of hsTn for impaired MAPSE implicating pre-defined cutoffs, and these models were adjusted for basic parameters (age, sex, creatinine) and biomarkers (NTproBNP and hsTn). Multivariable linear or logistic regression analyses were performed with backward elimination. Parameters in multivariable models were included to these models as independent variables, when they revealed a known clinical impact on or significant univariate correlations with the dependent variable. Data are presented as means with confidence intervals (CI) or medians with interquartile ranges (IQR) (25th to 75th percentiles), depending on the distribution of the data. *p* values < 0.05 were considered as statistically significant. Statistical analyses were performed in all patients and in three sub-groups according to MAPSE: (MAPSE I: ≥ 11 mm; MAPSE II: ≥ 8 mm − < 11 mm; MAPSE III: < 8 mm). Cutoffs of biomarkers were set at the group specific medians of each biomarker for the groups of reduced MAPSE. Power calculations were performed when multivariable regression models revealed lacking statistical significance for the tested biomarkers. The calculations were performed with GraphPad Prism software (GraphPad Software Inc., San Diego, CA, USA) and SPSS software (IBM SPSS Statistics, IBM Corp., Armonk, NY, USA).

## Results

### Study population

A total of 84 patients were enrolled in the present study. Median age of the patients was 55 years (range 18–85 years). Most patients were of male gender (n = 58, 69%). Twenty-six patients suffered from compensated CHF (according to LVEF < 55%) with only mild to moderate symptoms according to NYHA class I and II (n = 24, 92% of CHF patients). Thirteen patients suffered from AF, mostly paroxysmal AF (n = 8, 10%) (Table 1). Thirty-one patients suffered from valvular heart disease, mostly from mitral valve regurgitation (MR) (n = 15, 18%), followed by tricuspid (n = 10, 12%) and aortic valve regurgitation (n = 5, 6%). Thirteen patients suffered from mild MR (87% of all mitral valve regurgitation patients). Only one patient suffered from MR grade III and one patient from aortic valve stenosis grade III (data not shown). Twenty-six patients presented with CAD, and 23% of these already underwent aorto-coronary bypass (ACVB) surgery (n = 6). Seven patients suffered from chronic kidney disease, while no patient suffered from end-stage renal failure or was in need for hemodialysis. The median glomerular filtration rate (GFR) was 89 mL/min (IQR 75–101 mL/min). Prior drug therapy is seen also in Table 1.

**Table 1:** Baseline characteristics of study patients

Characteristic	Patients (n = 84)
Age, mean (range; 95% CI)	55 (18–85; 52–59)
Gender, n (%)	
Male	58 (69)
Female	26 (31)
Cardiovascular risk factors, n (%)	
Arterial hypertension	37 (44)
Hypercholesterinemia	21 (25)
Cardiac family history	15 (18)
Smoking status	32 (38)
Diabetes mellitus	11 (13)
Adipositas	12 (14)
Laboratory parameters, median (IQR)	
Creatinine (mg/dL)	0.89 (0.78–1.04)
GFR (mL/min)	89 (75–101)
Prior medical history, n (%)	
Chronic heart failure	26 (31)
NYHA I	10 (38)
NYHA II	14 (54)
NYHA III	2 (8)
NYHA IV	0 (0)
Atrial fibrillation	13 (15)
Paroxysmal	8 (10)
Persistent	3 (4)
Permanent	2 (2)
Coronary artery disease	26 (31)
1 vessel disease	10 (12)
2 vessel disease	3 (4)
3 vessel disease	13 (15)
Past history of myocardial infarction	17 (20)
Valvular heart disease	31 (37)
Chronic kidney disease	7 (8)
COPD	7 (8)
Asthma	6 (7)
Pneumonia	2 (2)
Pulmonary hypertension	1 (1)
Cancer	7 (8)
Medication, n (%)	
ACE-inhibitor/AT1-receptor antagonist	43 (51)
Beta blocker	43 (51)
Aldosterone antagonist	15 (18)
Calcium antagonist	18 (21)
Diuretics	45 (54)
Acetylsalicylic acid	32 (38)
Thienopyridines	9 (11)
OAC/NOAC	14 (17)
Statin	34 (40)

*ACE* angiotensin converting enzyme, *AT1* angiotensin 1, *OAC* oral anticoagulant, *NOAC* novel oral anticoagulant, *CI* confidence interval, *IQR* interquartile range

According to the extent of MAPSE, three subgroups were defined as follows: MAPSE I: MAPSE ≥ 11 mm (n = 35, 42%), MAPSE II: MAPSE ≥ 8 to < 11 mm (n = 31, 37%), MAPSE III: MAPSE < 8 mm (n = 18, 21%).

The presence of CHF NYHA III (NYHA IV patients were not included in the present study), three-vessel CAD, grade III valvular heart disease, permanent AF and chronic kidney disease were comparable between the subgroups. Cardiac alterations in MAPSE subgroups were as follows: CHF NYHA III: MAPSE I: 0%, II: 50%, III: 50%; three-vessel CAD: MAPSE I: 31%, II: 54%, III: 15%; grade III valvular heart disease: MAPSE I: 50%, II: 50%, III: 0%; permanent AF: MAPSE I: 0%, II: 33%, III: 67%; chronic kidney disease: MAPSE I: 14%, II: 43%, III: 43%.

### Distribution of cardiac MRI indices according to MAPSE subgroups

Median LVEF was 59% (IQR 51–64%) in the total cohort (Additional file 1: Table S1). LVEF decreased significantly according to impaired subgroups of MAPSE (*p* = 0.007). Despite the exclusion of patients with RV dysfunction (RVF < 50%), tricuspid annular plane systolic excursion (TAPSE) decreased significantly alongside with impaired MAPSE (*p* = 0.0001) (Additional file 1: Table S1). Furthermore, there were significant differences between between MAPSE subgroups regarding posterior wall thickness (PWT; *p* = 0.047) as well as LV stroke volume (LVSV; *p* = 0.0001), RV end-diastolic volume (RVEDV, *p* = 0.0001), RV end-systolic volume (RVESV, *p* = 0.0001) and RVSV (*p* = 0.0001) each being standardized with body surface area (BSA). No significant differences were observed for remodeling index, RVEF, septal wall thickness (SWT), LVEDV/BSA and LVESV/BSA in the subgroups of MAPSE (*p* ≥ 0.05) (Additional file 1: Table S1).

### Clinical confounding factors influencing MAPSE

Within multivariable linear regression models known clinical characteristics, cardiac comorbidities as well as hsTn were adjusted to evaluate their association on MAPSE within the present cohort. Adjusting for hsTn and the presence of CAD, valvular heart disease and AF, only hsTn and the presence of AF were associated significantly with MAPSE (*p* < 0.05) (Table 2).

**Table 2:** Multivariable linear regression models for evaluating associations between MAPSE and clinical characteristics and cardiac comorbidities

	Beta	*T*	Adjusted *p* value	Beta	*T*	Adjusted *p* value
CAD	− 0.06	− 0.51	0.62	− 0.10	− 0.92	0.36
Valvular heart diseases	− 0.10	− 0.97	0.34	− 0.06	− 0.58	0.56
Atrial fibrillation	− 0.23	− 2.18	*0.03*	− 0.27	− 2.65	*0.01*
Log hsTnT	− 0.31	− 2.96	*0.004*	–	–	–
Log hsTnI	–	–	–	− 0.27	− 2.61	*0.01*

*CAD* coronary artery disease

Italic values indicate statistically significant *p* values (*p* < 0.05)

### HsTn and NT-proBNP in MAPSE sub-groups

**Fig. 1:**
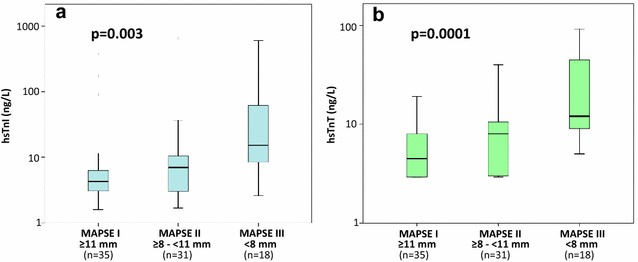
Distribution of hsTnI (**a**) and hsTnT (**b**) serum concentrations according to subgroups of reduced MAPSE. Data are presented as medians with 25th and 75th percentiles (boxes) and 5th and 95th percentiles (whiskers)

Figure 1a, b demonstrate significantly increasing hsTn values according to subgroups of decreased MAPSE (*p* = 0.003 and *p* = 0.0001). Due to the known left-skewed distribution of hsTn [20], a severe reduction of MAPSE (MAPSE III) results in disproportionately high values of hsTn, and thus in a high 95% confidence interval. Biomarker levels were as follows:

HsTnT: MAPSE I (median 5 ng/L, IQR 3–9 ng/L), MAPSE II (median 8 ng/L, IQR 3–11 ng/L), MAPSE III (median 12 ng/L, IQR 9–46 ng/L). HsTnI: MAPSE I (median 4.3 ng/L, IQR 3.1–6.4 ng/L); MAPSE II (median 7.0 ng/L, IQR 2.9–11.5 ng/L); MAPSE III (median 15.6 ng/L, IQR 8.1–62.8 ng/L). NT-proBNP (data not shown): MAPSE I (median 55.7 ng/L, IQR 31.3–134.9 ng/L); MAPSE II (median 151.8 ng/L, IQR 38.3–406.8 ng/L); MAPSE III (median 808.1 ng/L, IQR 229.4–2285.8 ng/L) (*p* = 0.0001).

### Correlations of hsTn with cMRI parameters and clinical characteristics

HsTn correlated significantly with MAPSE (hsTnI: *r* = − 0.33; *p* = 0.002; hsTnT: *r* = − 0.40; *p* = 0.0001), age (hsTnI: *r* = 0.45; *p* = 0.0001; hsTnT: *r* = 0.65; *p* = 0.0001) and NT-proBNP (hsTnI: *r* = 0.64; *p* = 0.0001; hsTnT: *r* = 0.69; *p* = 0.0001). Only hsTnT correlated significantly with creatinine (*r* = 0.24; *p* = 0.03), whereas hsTnI did not (*r* = 0.09; *p* = 0.4).

Additional file 2: Table S2 shows univariate correlations of hsTn with cMRI parameters in all patients. HsTn did not reveal significant correlations with LV functional parameters (except for MAPSE) or TAPSE. In contrast, hsTn significantly correlated with posterior and septal wall thickness, remodeling index, as well as RVEF, RV end-diastolic and end-systolic volumes indexed to BSA, despite the exclusion of patients with RV dysfunction (RVF < 50%).

Afterwards, clinical characteristics and cardiac comorbidities including MAPSE were adjusted within multivariable linear regression models to evaluate their association with hsTn. As shown in Table 3, MAPSE < 8 mm was correlated inversely with both hsTnI (left panel: Beta = − 0.19; *T* = − 1.96; *p* = 0.05) and hsTnT (right panel: Beta = − 0.26; *T* = − 3.26; *p* = 0.002) even after adjusting with age, sex, creatinine, the presence of CAD, valvular heart disease, AF and impaired LAF < 45% (backward stepwise analysis).

**Table 3:** Multivariable linear regression model for evaluating associations between hsTn and clinical characteristics and cardiac comorbidities

	Log hsTnI			Log hsTnT		
	Beta	*T*	Adjusted *p* value	Beta	*T*	Adjusted *p* value
Age	0.11	1.02	0.31	0.26	3.05	*0.003*
Sex	0.04	0.41	0.69	0.04	0.54	0.59
Creatinine	0.27	2.82	*0.006*	0.40	4.96	*0.0001*
CAD	0.31	3.35	*0.001*	0.21	2.53	*0.01*
Valvular heart diseases	− 0.15	− 1.59	0.12	− 0.07	− 0.89	0.38
Atrial fibrillation	− 0.11	− 0.99	0.32	− 0.11	− 1.19	0.24
LAF (< 45%)	− 0.23	− 2.36	*0.02*	− 0.11	− 1.33	0.19
MAPSE (< 8 mm)	− 0.19	− 1.96	0.05	− 0.26	− 3.26	*0.002*

*CAD* coronary artery disease, *LAF* left atrial function, *MAPSE* mitral annular plane systolic excursion

Italic values indicate statistically significant *p* values (*p* < 0.05)

### hsTn discriminate reduced MAPSE

**Fig. 2:**
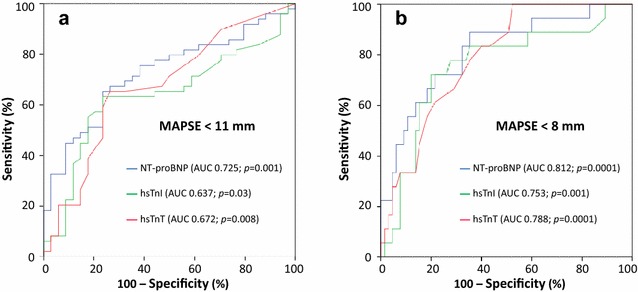
Receiver operating characteristic curves (ROC) revealing valuable discrimination of patients with reduced MAPSE of < 11 mm (**a**) and < 8 mm (**b**)

As analyzed by ROC curves, both hsTnT and hsTnI were able to discriminate significantly patients with impaired MAPSE < 11 mm from all others (hsTnT: AUC = 0.67; 95% CI 0.55–0.79; *p* = 0.008; hsTnI: AUC = 0.64; 95% CI 0.52–0.76; *p* = 0.03) (Fig. 2a). Furthermore, both hsTnT and hsTnI were able to discriminate significantly patients with reduced MAPSE < 8 mm from all others (hsTnT: AUC = 0.79; 95% CI 0.69–0.89; *p* = 0.0001; hsTnI: AUC = 0.75; 95% CI 0.62–0.89; *p* = 0.001) (Fig. 2b). In contrast, AUCs of NT-proBNP were numerically greater than the AUCs of hsTn (NT-proBNP: MAPSE < 11 mm: AUC = 0.73; 95% CI 0.62–0.83; *p* = 0.001; MAPSE < 8 mm: AUC = 0.81; 95% CI 0.70–0.93; *p* = 0.0001) but did not reveal a significant difference (*p* > 0.05) when comparing these ROC curves by the method of Hanley et al. [19] (Fig. 2a, b).

### hsTn reveal independent associations with impaired MAPSE

Finally, clinical characteristics, NT-proBNP and hsTn were adjusted within multivariable logistic regression models to evaluate their association with impaired MAPSE.

As shown in Table 4, patients with increased hsTnI levels ≥ 8.4 ng/L were 4.2 times more likely to suffer from MAPSE < 11 mm (left panel: adjusted odds ratio (OR) = 4.22; 95% CI 1.43–12.42; *p* = 0.009), whereas patients with hsTnT values ≥ 9 ng/L were 4.7 times more likely to suffer from MAPSE < 11 mm (right panel: OR = 4.71; 95% CI 1.78–12.51; *p* = 0.002), even after adjustment with age, sex, creatinine and NT-proBNP (p > 0.05).

**Table 4:** Multivariable logistic regression models for evaluating the diagnostic ability of hsTn to identify patients with reduced MAPSE of < 11 mm

	hsTnI			hsTnT		
	Adjusted odds ratio	95% CI	Adjusted *p* value	Adjusted odds ratio	95% CI	Adjusted *p* value
Age	0.97	0.94–1.00	*0.048*	0.99	0.95–1.02	0.42
Sex^a^	2.44	0.81–7.33	0.11	2.19	0.76–6.28	0.15
Creatinine	0.47	0.04–5.76	0.56	0.54	0.06–4.78	0.58
NT-proBNP (≥ 285.2 ng/L)	1.81	0.45–7.30	0.40	2.48	0.66–9.34	0.18
hsTnI (≥ 8.4 ng/L)	4.22	1.43–12.42	*0.009*	–	–	–
hsTnT (≥ 9 ng/L)	–	–	–	4.71	1.78–12.51	*0.002*

*CI* confidence interval

Italic values indicate statistically significant *p* values (*p* < 0.05)

^a^An adjusted odds ratio of < 1 indicates an association of female gender with reduced MAPSE

In contrast, patients with hsTnI levels ≥ 15.55 ng/L were not significantly associated with MAPSE < 8 mm (*p* = 0.28), whereas patients with hsTnT values ≥ 12 ng/L were 3 times more likely to suffer from MAPSE < 8 mm (right panel: OR = 3.42; 95% CI 0.99–11.79; *p* = 0.05) in models being adjusted with age, sex, creatinine and NT-proBNP (Table 5). Noteworthy, increased NT-proBNP levels were significantly associated with severely reduced MAPSE < 8 mm (*p* < 0.005). However, statistical power analysis in this subgroup revealed a valuable statistical power above 90% for hsTnI (difference between means of 0.441 (1.355–0.9136, mean log10 hsTnI MAPSE III—mean log10 hsTnI MAPSE I + II) *p* < 0.05). For hsTnT, the power was slightly lower with 70% to detect a difference between means of 0.021 with a significance level (alpha) of 0.05 (two-tailed), while 50 patients in each group were needed to get a power of 80%.

**Table 5:** Multivariable logistic regression models for evaluating the diagnostic ability of hsTn to identify patients with reduced MAPSE of < 8 mm

	hsTnI			hsTnT		
	Adjusted odds ratio	95% CI	Adjusted *p* value	Adjusted odds ratio	95% CI	Adjusted *p* value
Age	0.98	0.94–1.02	0.36	0.99	0.95–1.04	0.71
Sex^a^	1.69	0.47–6.08	0.42	1.63	0.46–5.79	0.45
Creatinine	0.84	0.22–3.25	0.80	1.02	0.28–3.80	0.97
NT-proBNP (≥ 808.0 ng/L)	10.00	2.87–34.84	*0.0001*	6.73	1.79–25.22	*0.005*
hsTnI (≥ 15.55 ng/L)	2.22	0.53–9.35	0.28	–	–	–
hsTnT (≥ 12 ng/L)	–	–	–	3.42	0.99–11.79	0.05

*CI* confidence interval

Italic values indicate statistically significant *p* values (*p* < 0.05)

^a^An adjusted odds ratio of < 1 indicates an association of female gender with reduced MAPSE

## Discussion

The present study demonstrates that levels of hsTn are able to reflect MAPSE being assessed by cMRI. Both hsTnI and hsTnT were inversely correlated with MAPSE within multivariable linear regression models and increased significantly according to the different stages of impaired MAPSE. HsTn discriminated both patients with impaired MAPSE < 11 and < 8 mm. In multivariable logistic regression models, hsTn were still significantly associated with impaired MAPSE < 11 mm, even after adjustment for known influencing factors on hsTn release being known in clinical routine [20, 21]. NT-proBNP revealed comparable associations with reduced MAPSE.

There are several proteins participating in the regulation of cardiac contraction such as the inhibitory protein TnI, the calcium binding protein TnC and the tropomyosin binding protein TnT, which together form the cTn complex. TnT is attached to the cardiac myofibrillar troponin–tropomyosin complex. TnC affinity for calcium is reduced by TnI, whereby troponin–tropomyosin interaction is inhibited [21]. Since both TnI and TnT are not expressed by damaged skeletal muscle, they are specific for cardiac injury [21–23]. cTn play an essential role in the diagnosis of acute myocardial infarction [10]. Furthermore, an association between elevated hsTn and an increased incidence rate of AF in patients with atherosclerosis was shown [24]. Besides, hsTnT correlated significantly with reduced LVEF as well as elevated LV end-diastolic pressure in patients with stable CHF [14]. Additionally, hsTn were associated with short- and long-term prognosis in CHF patients [7, 12, 13]. Dinh et al. demonstrated an association of hsTnT and HFpEF, whereas the association is proportional to the severity of the disease [12]. Tsutamoto et al. showed that hsTnI is an independent prognostic predictor in patients with CHF [13]. Furthermore, temporal increases of hsTnT were independently associated with incident coronary heart disease, death, and HF events [25]. Several studies demonstrated a correlation between elevated cTn levels and an increased risk of morbidity and mortality in both acute HF and CHF [21].

HF represents a preventable and treatable disease [26]. In early stages of HF, patients can present with asymptomatic structural or functional cardiac alterations (systolic or diastolic LV dysfunction, reduced MAPSE), which are known as precursors of HF. It is of major importance to identify these precursors because they are related to a poor outcome. Starting clinical follow-up as well as targeted medical therapy at the precursor stages of HF may reduce mortality in patients with asymptomatic compensated HF [27, 28].

Myocardial contraction during systole equates a combination of longitudinal, radial and circumferential contractions [2, 6] resulting in a combination of long axis shortening, radial wall thickening and circumferential shortening [29]. Whereas LVEF only reflects the combined function of all components, but never specifically assesses longitudinal function, MAPSE represents the amount of displacement of the mitral annular plane towards the apex [4], and thus particularly reflects the contraction of longitudinal fibers, the corresponding longitudinal function [3, 4]. In diverse cardiac pathologies, transformations of longitudinal function already appear, when radial and circumferential functions are still unaffected. Accordingly, longitudinal dysfunction assessed by MAPSE appears to be an early marker for cardiac pathologies [3, 4, 6, 29]. It was demonstrated that MAPSE was able to detect more subtle abnormalities of LV function (LVF) than LVEF [30, 31]. Wenzelburger et al. showed that MAPSE could identify patients with HF and impaired LVF even when LVEF is still normal [3].

Longitudinal function plays an important role in cardiac mechanics [29]. During LV systole, the mitral annulus is pulled towards the apex as a result of long-fiber contraction, causing longitudinal shortening and a reduction of cavity size of the ventricle [2, 6, 32]. This longitudinal shortening contributes 60% to the normal stroke volume [33, 34]. During early LV diastole, the position of the mitral annulus creates ventricular suction and thus ventricular filling [29, 34]. During advanced LV diastole, the mitral annulus moves back away from the apex, causing ventricular filling by moving around the column of blood stored in the left atrium [2, 6, 29, 34].

MAPSE was shown to correlate with global systolic LVF [4]. Furthermore, MAPSE correlated with several factors affecting LVF such as AF, CAD, myocardial infarction, dilated cardiomyopathy, HF and age [29, 30, 35–37]. Willenheimer et al. showed that MAPSE was strongly related to 1-year mortality [35]. Furthermore, Sveälv et al. found out that long-axis function had a significant influence on 10-year survival in patients with CHF [38]. MAPSE was shown to be an independent predictor of major adverse cardiovascular events [29]. The main findings of Bergenzaun et al. were that MAPSE was an independent predictor of 28-day mortality in critically ill patients with shock and systemic inflammation [39]. Furthermore, Rydberg et al. suggested that reduced MAPSE, but not LVEF, is an independent predictor for the degree of aortic stenosis [40]. In several studies, a correlation between MAPSE and LVEF was demonstrated [2, 41]. Especially a value of MAPSE of less than 8 mm was associated with a reduced LVEF (< 50%) with a specificity of 82% and a sensitivity of 98% [42].

The major advantage of the method assessing MAPSE is the simplicity of its measurement [43]. In the majority of patients, MAPSE can be assessed quite independent of imaging quality [4]. It requires no special analysis software, can be performed rapidly and shows good inter- and intra-observer variability [18, 29]. In cMRI-derived MAPSE measurements, even better intra-and inter-observer reproducibility compared to previous echocardiography studies were obtained [18].

To the best of our knowledge, the present study is the first, investigating the correlation between hsTnT, hsTnI and MAPSE being assessed by cMRI. Only one prior study suggested an association between hsTnT and MAPSE assessed via transthoracic echocardiographic examination in 47 critically ill patients with shock [39]. However, this analysis aims to combine the assessment of MAPSE by modern cMRI with the combination of two different pathophysiological families of blood biomarkers, the natriuretic peptide NT-proBNP and the ischemic biomarkers hsTnT and hsTnI.

Therefore, the diagnostic combination of hsTn and MAPSE being assessed by cMRI might detect potentially early stages of LV dysfunction as being indicated by impaired MAPSE already, despite a still preserved or only slightly reduced LVEF and beyond clinical confounding factors known to influence hsTn release such as age, AF, renal failure. This sets out hsTn as even more sensitive biomarkers being of valuable benefit for realistic clinical settings. Detecting LV dysfunction already at very early and compensated stages of CHF with only mild to moderate symptoms revealing only mild to moderate reduction of LVEF and a still preserved LVSV turns MAPSE into a precursor of HF. Whether routine measurements of hsTn either with or without cMRI imaging within standard of care in such patients might lead to a more close-meshed follow-up of these patients or might initiate or improve HF treatment at earlier stages needs to be addressed in upcoming larger prospective and randomized controlled studies.

## Study limitations

Statistical power might have been only moderate in the subgroup of patients with MAPSE < 8 mm to specifically confirm/exclude an additional diagnostic value for hsTnT, as seen in the lack of statistical significance in multivariable regressions. Therefore, the present results need to be confirmed within larger patient cohorts including similar patients evaluating both hsTnT and hsTnI.

## Additional files

**Additional file 1: Table S1.** Distribution of cardiac MRI indices according to MAPSE subgroups.

**Additional file 2: Table S2.** Univariate correlations between hsTn and cardiac MRI parameters in all patients (*n* = 84).
