# Correction to: High sensitivity troponin T and I reflect mitral annular plane systolic excursion being assessed by cardiac magnetic resonance imaging

**DOI:** 10.1186/s40001-018-0306-0

**Published:** 2018-02-19

**Authors:** Michèle Natale, Michael Behnes, Seung-Hyun Kim, Julia Hofmann, Nadine Reckord, Ursula Hofmann, Johannes Budjan, Siegfried Lang, Martin Borggrefe, Theano Papavassiliu, Thomas Bertsch, Ibrahim Akin

**Affiliations:** 1 First Department of Medicine, Faculty of Medicine Mannheim, University Medical Center Mannheim (UMM), University of Heidelberg, Theodor-Kutzer-Ufer 1-3, 68167 Mannheim, Germany; 2 Institute of Clinical Radiology and Nuclear Medicine, Faculty of Medicine Mannheim, University Medical Center Mannheim (UMM), University of Heidelberg, Mannheim, Germany; 3 Institute of Clinical Chemistry, Laboratory Medicine and Transfusion Medicine, General Hospital Nuremberg, Paracelsus Medical University, Nuremberg, Germany

## Correction to: Eur J Med Res (2017) 22:38 10.1186/s40001-017-0281-x

Following publication of the original article [1], the authors reported that there was a mistake in the Methods section → Measurements of biomarkers (page 6, line 3): 2000*g* should read 2500*g*. So, the correct sentence should be “All samples were obtained by venipuncture into serum monovettes^®^ and centrifuged at 2500*g* for 10 min at 20 °C.”
