# Supplementary material for: High sensitivity troponin T and I reflect mitral annular plane systolic excursion being assessed by cardiac magnetic resonance imaging
Source: Eur J Med Res. 2017 Oct 4;22:38. doi: 10.1186/s40001-017-0281-x (PMC5628434; doi:10.1186/s40001-017-0281-x)
Supplement: Supplementary file 1 — Additional file 1: Table S1. Distribution of cardiac MRI indices according to MAPSE subgroups. [file 40001_2017_281_MOESM1_ESM.doc]

| **Table S1. Distribution of cardiac MRI indices according to MAPSE subgroups** | | | | | |
| --- | --- | --- | --- | --- | --- |
|  | All patients  (n=84) | MAPSE I  ≥ 11 mm  (n = 35) | MAPSE II  ≥ 8 - < 11 mm  (n = 31) | MAPSE III  < 8 mm  (n = 18) | *p*-value |
| LVEF | 59.00  (51.00 - 64.00) | 61.00  (56.00 - 66.00) | 57.00  (45.00 - 61.00) | 57.00  (36.00 - 60.50) | **0.007** |
| LVEDV/BSA ^a^ | 85.16  (74.02 - 100.82) | 91.66  (80.08 - 103.19) | 82.94  (66.46 - 92.15) | 82.42  (68.04 - 105.28) | 0.05 |
| LVESV/BSA ^a^ | 36.03  (26.37 - 47.44) | 36.05  (27.71 - 42.14) | 36.00  (25.30 - 50.02) | 34.78  (24.81 - 65.08) | 0.76 |
| LVSV/BSA ^a^ | 48.48  (39.98 - 55.22) | 55.00  (48.80 - 61.88) | 44.05  (38.74 - 50.55) | 38.68  (33.52 - 49.86) | **0.0001** |
| RVEF | 62.35  (56.14 - 66.43) | 61.89  (55.42 - 64.67) | 61.46  (56.07 - 66.92) | 64.12  (57.10 - 66.90) | 0.51 |
| RVEDV/BSA ^a^ | 74.35  (64.62 - 87.53) | 86.39  (79.25 - 94.19) | 66.16  (59.85 - 76.50) | 66.05  (44.11 - 75.32) | **0.0001** |
| RVESV/BSA ^a^ | 28.62  (22.18 - 37.04) | 33.77  (28.66 - 39.18) | 25.81  (20.20 - 30.46) | 23.93  (15.23 - 33.05) | **0.0001** |
| RVSV/BSA ^a^ | 45.67  (38.92 - 52.61) | 52.61  (45.60 - 59.21) | 41.57  (35.47 - 46.50) | 39.72  (28.89 - 49.24) | **0.0001** |
| TAPSE | 1.90  (1.48 - 2.23) | 2.16  (1.89 - 2.41) | 1.68  (1.45 - 2.05) | 1.42  (0.78 - 2.00) | **0.0001** |
| Remodeling index | 0.80  (0.68 - 0.92) | 0.78  (0.65 - 0.90) | 0.79  (0.70 - 0.89) | 0.97  (0.76 - 1.18) | 0.06 |
| PWT | 7.00  (6.00 - 8.00) | 7.00  (6.00 - 8.00) | 6.00  (6.00 - 7.00) | 8.00  (6.00 - 9.00) | 0.05 |
| SWT | 10.00  (8.00 - 12.00) | 10.00  (8.00 - 12.00) | 9.00  (8.00 - 11.00) | 10.00  (8.00 - 14.25) | 0.59 |
| LVEF, left ventricular ejection fraction; LVEDV, LV enddiastolic volume; LVESV, LV endsystolic volume; LVSV, LV stroke volume; RVEF, right ventricular ejection fraction; RVEDV, RV enddiastolic volume; RVESV, RV endsystolic volume; RVSV, RV stroke volume; TAPSE, tricuspid annular plane systolic excursion; PWT, posterior wall thickness; SWT, septal wall thickness.  ^a^ Body surface area  Data presented as median with interquartile ranges (IQR).  Bold values indicate statistically significant *p* values (*p* < 0.05). | | | | | |
