# Supplementary material for: High sensitivity troponin T and I reflect mitral annular plane systolic excursion being assessed by cardiac magnetic resonance imaging
Source: Eur J Med Res. 2017 Oct 4;22:38. doi: 10.1186/s40001-017-0281-x (PMC5628434; doi:10.1186/s40001-017-0281-x)
Supplement: Supplementary file 2 — Additional file 2: Table S2. Univariate correlations between hsTn and cardiac MRI parameters in all patients (n = 84). [file 40001_2017_281_MOESM2_ESM.doc]

| **Table S2. Univariable correlations between hsTn and cardiac MRI parameters in all patients (n=84)** | | | | | | |
| --- | --- | --- | --- | --- | --- | --- |
|  | **hsTnI** | |  | | **hsTnT** | |
|  | *r* | *p* value | | *r* | | *p* value |
| LVEF | - 0.16 | 0.14 | | - 0.21 | | 0.06 |
| LVEDV/BSA ^a^ | 0.13 | 0.26 | | 0.05 | | 0.67 |
| LVESV/BSA ^a^ | 0.15 | 0.18 | | 0.14 | | 0.22 |
| LVSV/BSA ^a^ | - 0.08 | 0.47 | | - 0.20 | | 0.07 |
| MAPSE | - 0.33 | **0.002** | | - 0.40 | | **0.0001** |
| RVEF | 0.30 | **0.005** | | 0.28 | | **0.01** |
| RVEDV/BSA ^a^ | - 0.28 | **0.01** | | - 0.37 | | **0.001** |
| RVESV/BSA ^a^ | - 0.39 | **0.0001** | | - 0.42 | | **0.0001** |
| RVSV/BSA ^a^ | - 0.11 | 0.33 | | - 0.21 | | 0.06 |
| TAPSE | - 0.05 | 0.63 | | - 0.12 | | 0.29 |
| Remodeling Index | 0.22 | **0.04** | | 0.22 | | 0.05 |
| PWT | 0.38 | **0.0001** | | 0.30 | | **0.006** |
| SWT | 0.34 | **0.002** | | 0.36 | | **0.001** |
| LVEF, left ventricular ejection fraction; LVEDV, LV enddiastolic volume; LVESV, LV endsystolic volume; LVSV, LV stroke volume; MAPSE, mitral annular plane systolic excursion; RVEF, right ventricular ejection fraction; RVEDV, RV enddiastolic volume; RVESV, RV endsystolic volume; RVSV, RV stroke volume; TAPSE, tricuspid annular plane systolic excursion; PWT, posterior wall thickness; SWT, septal wall thickness.  ^a^ Body surface area  Bold values indicate statistically significant *p* values (*p* < 0.05) | | | | | | |
